# A multivariate neuromonitoring approach to neuroplasticity-based computerized cognitive training in recent onset psychosis

**DOI:** 10.1038/s41386-020-00877-4

**Published:** 2020-10-07

**Authors:** Shalaila S. Haas, Linda A. Antonucci, Julian Wenzel, Anne Ruef, Bruno Biagianti, Marco Paolini, Boris-Stephan Rauchmann, Johanna Weiske, Joseph Kambeitz, Stefan Borgwardt, Paolo Brambilla, Eva Meisenzahl, Raimo K. R. Salokangas, Rachel Upthegrove, Stephen J. Wood, Nikolaos Koutsouleris, Lana Kambeitz-Ilankovic

**Affiliations:** 1 Department of Psychiatry, Icahn School of Medicine at Mount Sinai, New York, NY USA; 2 Department of Psychiatry and Psychotherapy, Ludwig-Maximilian University, Munich, Germany; 3 Department of Education, Psychology, Communication – University of Bari “Aldo Moro”, Bari, Italy; 4 University of Cologne, Faculty of Medicine and University Hospital of Cologne, Cologne, Germany; 5 Department of R&D, Posit Science Corporation, San Francisco, CA USA; 6 Department of Pathophysiology and Transplantation, Faculty of Medicine and Surgery, University of Milan, Milan, Italy; 7 Department of Radiology, University Hospital, Ludwig-Maximilian University, Munich, Germany; 8 Translational Psychiatry Unit (TPU), Department of Psychiatry and Psychotherapy, University of Luebeck, Luebeck, Germany; 9 Department of Neuroscience and Mental Health, Fondazione IRCCS Ca’ Granda Ospedale Maggiore Policlinico, Milan, Italy; 10 Department of Pathophysiology and Mental Health, University of Milan, Milan, Italy; 11 Department of Psychiatry and Psychotherapy, Medical Faculty, Heinrich-Heine University, Düsseldorf, Germany; 12 Department of Psychiatry, University of Turku, Turku, Finland; 13 School of Psychology, University of Birmingham, Birmingham, United Kingdom; 14 Institute of Mental Health, University of Birmingham, Birmingham, United Kingdom; 15 Orygen, the National Centre of Excellence for Youth Mental Health, Melbourne, VIC Australia; 16 Centre for Youth Mental Health, University of Melbourne, Melbourne, VIC Australia

**Keywords:** Predictive markers, Psychosis

## Abstract

Two decades of studies suggest that computerized cognitive training (CCT) has an effect on cognitive improvement and the restoration of brain activity. Nevertheless, individual response to CCT remains heterogenous, and the predictive potential of neuroimaging in gauging response to CCT remains unknown. We employed multivariate pattern analysis (MVPA) on whole-brain resting-state functional connectivity (rsFC) to (neuro)monitor clinical outcome defined as psychosis-likeness change after 10-hours of CCT in recent onset psychosis (ROP) patients. Additionally, we investigated if sensory processing (SP) change during CCT is associated with individual psychosis-likeness change and cognitive gains after CCT. 26 ROP patients were divided into maintainers and improvers based on their SP change during CCT. A support vector machine (SVM) classifier separating 56 healthy controls (HC) from 35 ROP patients using rsFC (balanced accuracy of 65.5%, *P* < 0.01) was built in an independent sample to create a naturalistic model representing the HC-ROP hyperplane. This model was out-of-sample cross-validated in the ROP patients from the CCT trial to assess associations between rsFC pattern change, cognitive gains and SP during CCT. Patients with intact SP threshold at baseline showed improved attention despite psychosis status on the SVM hyperplane at follow-up (*p* < 0.05). Contrarily, the attentional gains occurred in the ROP patients who showed impaired SP at baseline only if rsfMRI diagnosis status shifted to the healthy-like side of the SVM continuum. Our results reveal the utility of MVPA for elucidating treatment response neuromarkers based on rsFC-SP change and pave the road to more personalized interventions.

## Introduction

Neuroplasticity-based computerized cognitive training (CCT) has frequently been used as a supplementary treatment in psychotic illness [1, 2]. CCT implements learning-based neuroplasticity principles to restore neuromodulatory processes underlying the structure, function, and connections in the brain that support perceptual, cognitive, social, and motor abilities often disturbed in psychotic illness [3, 4]. This therapeutic approach received evidence in circumventing cognitive deficits [5–7] and poor functional outcome in psychosis [8, 9]. Previous meta-analyses indicate that cognitive remediation has a small to moderate effect on multiple cognitive domains including attention, working memory, executive functioning, and social cognition in the treatment of schizophrenia [6, 7, 10]. In particular, research has documented the neural plasticity of cortical responses as an individual acquires new perceptual and cognitive abilities [11, 12]. Further evidence suggests that preserved brain network modularity [13] and neuronal fiber integrity may be important determinants for training-induced neurocognitive plasticity, particularly in domains of attention [14], executive function [14], and social cognition [15]. Previous research on selective attention demonstrates marked malleability of neural systems in charge of potential changes in response to intervention [16]. Dysplasticity in schizophrenia has been known for decades, and while it has predominantly been reported in motor and frontal areas [17, 18], it is also expressed in multiple brain regions including sensory systems [19]. The underlying mechanism of neuroplasticity-based CCT is meant to induce widespread changes in both cortical and subcortical representations and may not be captured by single-region activation maps measured by task-based MRI [3, 20, 21].

Importantly, the variability in neuroplastic response induced by intermediate neurocognitive and brain phenotypes may moderate the neuroplastic response induced by respective training paradigms [22]. To mitigate the heterogeneity in response to CCT and multidimensionality of neuroimaging data, multivariate pattern analysis (MVPA) allows quantification of diagnostic group membership or treatment response at the individual level [23, 24], particularly when clinical data is complemented with neurobiological proxies [25]. These proxies may entail information on intermediate- and endo-phenotypes responsible for the high degree of variability in the response to CCT. Specifically, they may serve as “neuromarkers” [26, 27] that successfully aid in identifying disorders and factors determining not only illness progression [28, 29], but also monitoring response to treatment (theranostics) [27, 30–32]. Recently, brain connectivity measures derived from task-based functional Magnetic Resonance Imaging (fMRI) were used as a proxy for cognitive performance [33]. Resting-state functional connectivity (rsFC) has been used to predict diagnosis and clinical outcome of patients with psychosis and it demonstrated a high level of within-subject reproducibility that is relevant for longitudinal monitoring of treatment response [34, 35].

Finally, the high degree of variability in cognitive gains may be explained by individual differences in engagement level of the underlying neural system target and learning progress in CCT [36, 37]. These studies showed greater deficits in mismatch negativity, an event-related potential elicited pre-attentively, predicted greater improvements after auditory CCT. Still, it remains unknown whether inter-individual differences in sensory processing during CCT in combination with neuroimaging prediction on the single-subject level may inform more personalized CCT in patients at the earlier stages of psychosis [38] early in the course of CCT (first 10 h).

The aim was to investigate individual response to 10 h of CCT by measuring changes in psychosis-likeness based on rsFC patterns in relation to sensory processing. First, we developed an original multivariate model, able to distinguish HC from ROP patients using rsFC in a naturalistic sample. Second, this model was applied to the CCT intervention sample, to assess and monitor clinical outcome in response to CCT. Hereby, we measured the change of psychosis-likeness after 10 h of CCT at the single-subject level employing machine learning on rsFC pattern before and after CCT. In the third step, we investigated how psychosis-likeness change was related to sensory processing. In the final step, we investigated the effects of sensory processing change (SPC), psychosis-likeness change (ROP-HC continuum) and their association on cognitive gains, in response to the intervention. We expected to observe cognitive gains in lower-order cognitive functions due to the drill-and-practice approach used and short duration of the intervention.

## Materials and methods

### Sample

Two samples were included from the Early Detection and Intervention Center at the Department of Psychiatry and Psychotherapy of the Ludwig-Maximilians-University (LMU) in Munich, Germany: (1) the original PRONIA study diagnostic sample of 35 ROP patients and 56 HC recruited from the LMU Munich site of the naturalistic, European multi-center PRONIA study [39] (Table 1) to generate the SVM classification HC-ROP model to create the psychosis-likeness hyperplane, and (2) the CCT intervention sample, independent from the original SVM sample cohort, that included 26 patients with ROP (Fig. S1) undergoing CCT in a randomized controlled trial (ClinicalTrials.gov Identifier: NCT03962426). Although PRONIA is a multi-center study, we included only the LMU, Munich site to generate our HC-ROP model as (1) the intervention sample was acquired from the same study site (2) neuroimaging site-effects can be an additional source of variability in SVM classification which is challenging to mitigate, especially for the resting-state modality [40–44]. For both the diagnostic classification and intervention samples, ROP patients were included if illness duration was below 2 years and if the criteria for an affective or non-affective psychotic episode according to the Diagnostic and Statistical Manual of Mental Disorders (DSM-IV) [45] was fulfilled (supplementary information, Section 1.1). All participants provided written informed consent prior to study inclusion while all procedures performed in this study were in accordance with the ethical standards of the Local Research Ethics Committee of the LMU and with the 1964 Helsinki Declaration and its later amendments or comparable ethical standards.

**Table 1:** Baseline demographic and clinical characteristics for ROP patients and HC individuals included for the generation of a healthy-to-psychosis model based on resting-state functional connectivity.

	ROP (*N* = 35)	HC (*N* = 56)	*T*/ χ^2^	*P* value
Number of female (%)	13 (37.14 %)	36 (64.29 %)	6.39	0.012*
Age (*SD*)	30.43 (6.15)	30.64 (6.78)	0.151	0.88
Years education (*SD*)^a^	13.88 (3.45)	15.73 (3.26)	2.51	0.014*
Premorbid IQ (*SD*)	100.29 (18.59)	109.64 (13.24)	2.80	0.006**
Handedness^a^	–	–	0.27	0.88
Right (%)	29	47	–	–
Mixed (%)	2	5	–	–
Left (%)	2	3	–	–
Diagnosis (%)				
No Axis I Diagnosis	0	56	–	–
Schizophrenia	19 (54.29 %)	–	–	–
Schizoaffective disorder	1 (2.63 %)	–	–	–
Schizophreniform disorder	3 (8.57 %)	–	–	–
Delusional disorder	5 (13.16 %)	–	–	–
Psychotic disorder NOS	5 (13.16 %)	–	–	–
Substance-induced psychotic disorder	2 (5.26 %)	–	–	–
GAF past month	41.18 (9.87)	83.7 (5.11)	26.91	<0.001***
GF current				
Role (*SD*)	5.06 (1.82)	8.29 (0.59)	12.24	<0.001***
Social (*SD*)	5.65 (1.32)	8.25 (0.69)	12.24	<0.001***
PANSS				
Total (SD)	67.03 (14.45)	–	–	–
Positive (SD)	18.00 (5.48)	–	–	–
Negative (SD)	15.06 (5.82)	–	–	–
General (SD)	33.97 (6.76)	–	–	–

*MRI* Magnetic Resonance Imaging, *NOS* not otherwise specified, *MDD* Major Depressive Disorder, *CPZ* chlorpromazine equivalent, *GAF* Global Assessment of Functioning, *GF* Global Functioning, *PANSS* Positive and Negative Syndrome Scale.

^a^Two participants did not provide total years of education at baseline and three did not complete the self-rating instrument which includes information regarding handedness.

### Procedures

#### CCT Intervention

Participants included in the active intervention group (*N* = 26, Table 2) completed an average of 9.98 h of CCT within 20 30-min individual sessions over 5 weeks (Supplementary Information, Fig. S1 and Section 1.2). The training consisted of four exercises (Table S1) that strike a balance in improving multiple cognitive domains including social cognition, processing speed, and attention. Task difficulty is adjusted to maintain 75–80% accuracy of the participants’ responses by constantly adapting presentation times of the displayed facial stimulus [3, 46]. Difficulty levels are modulated based on a specific individual’s rate of learning, represented by a ‘learning score’, are quantified by analyzing the stimulus presentation times for a specific level within a specific task (Supplementary Information, Section 1.3) and have previously been shown to influence neural plasticity and transfer of the training [47]. While all four exercises target early social sensory processing, we chose to study the Emotion Matching Task (EMT) as a potential proxy for target engagement, given its ability to capture the processing of basic social information while improving speeded facial emotion decision-making (Supplementary Information, Section 1.3). 26 patients that completed training on the Emotion Matching Task (EMT) were thus dichotomized into maintainers (*N* = 14) and improvers (*N* = 12) based on a median split of their learning scores (Supplementary Information, Section 1.3, Fig. S2). Improvers showed impaired performance at baseline and reached the psychophysical threshold (~31 ms) for EMT during training (high SPC), while maintainers showed intact psychophysical threshold for EMT at baseline that were sustained throughout the training (low SPC). The current analysis selected a level that was played by everyone and contained the most repetitions per participant.

**Table 2:** Baseline demographic information of the intervention sample.

	Maintainers EMT (*N* = 14)	Improvers EMT (*N* = 12)	*T*/ χ^2^	*P* value
Number of female (%)	8 (57.14%)	3 (25.00%)	2.74	0.098
Age (*SD*)	27.46 (5.84)	26.10 (7.00)	0.54	0.594
Years education (*SD*)	14.96 (2.71)	15.79 (4.73)	−0.56	0.582
Premorbid IQ (*SD*)	97.14 (16.02)	100.83 (13.62)	−0.63	0.537
Handedness	–	–	2.20	0.333
Right (%)	9	11	–	–
Mixed (%)	2	0	–	–
Left (%)	1	1	–	–
Diagnosis	–	–	6.55	0.477
Schizophrenia (%)	4 (28.57 %)	4 (33.33 %)	–	–
Schizoaffective disorder (%)	1 (7.14 %)	-	–	–
Schizophreniform disorder (%)	1 (7.14 %)	2 (16.67 %)	–	–
Brief psychotic disorder (%)	3 (21.43 %)	3 (25.00 %)	–	–
Delusional disorder (%)	1 (7.14 %)	2 (16.67 %)	–	–
Psychotic disorder NOS (%)	1 (7.14%)	–	–	–
MDD with psychotic symptoms (%)	3 (21.43 %)	–	–	–
Substance-induced psychotic disorder (%)	–	1 (8.33 %)	–	–
Medication at baseline (*N* = 39)				
CPZ equivalent (SD)	142.68 (162.49)	278.44 (258.96)	−1.63	0.117
Days between assessments	51.29 (13.12)	47.42 (8.99)	0.86	0.397
Number of hours trained	9.91 (0.74)	10.10 (0.73)	−0.49	0.630
GAF past month	46.25 (13.86)	48.00 (16.87)	−0.29	0.774
GF current				
Role (*SD*)	4.57 (1.45)	4.25 (1.54)	0.55	0.590
Social (*SD*)	6.00 (1.30)	6.00 (0.95)	0.00	1.000
PANSS				
Total (SD)	66.07 (15.61)	69.83 (17.94)	−0.57	0.573
Positive (SD)	19.21 (6.12)	19.83 (5.88)	−0.26	0.796
Negative (SD)	13.43 (5.24)	15.83 (6.19)	−1.07	0.294
General (SD)	33.43 (9.10)	34.17 (9.11)	−0.21	0.839

*EMT* Emotion Matching Task, *MRI* Magnetic Resonance Imaging, *NOS* not otherwise specified, *MDD* Major Depressive Disorder, *CPZ* chlorpromazine equivalent, *GAF* Global Assessment of Functioning, *GF* Global Functioning, *PANSS* Positive and Negative Syndrome Scale.

### Assessment procedure

Clinical assessment occurred during intake at baseline (T0) and again at follow-up (FU) post-intervention. Clinical diagnosis was assessed using the Structured Clinical Interview for Diagnostic and Statistical Manual of Mental Disorders (SCID) [45]. In order to assess clinical status and the presence and severity of symptoms, the Positive and Negative Syndrome Scale (PANSS) was administered [48]. Global rating of functioning was assessed using the Global Assessment of Functioning (GAF) Disability and Impairment Scale of the DSM-IV [49]. Additionally, the clinician-rated Global Functioning - Social (GF-S) and Global Functioning - Role (GF-R) Scales were used to assess social and role functioning separately [50].

A cross-domain neuropsychological test battery comprising 9 tests were administered to patients in the intervention sample at T0 and FU in a fixed order (Supplementary Information, Section 1.4). Tests were z-score transformed based on the study sample to closely reflect cognitive domains based on the Measurement and Treatment Research to Improve Cognition in Schizophrenia (MATRICS) recommended procedures [51] (Table S2).

### Imaging procedure

All participants from both the original sample and intervention sample were scanned using the same 3 Tesla Philips Ingenia scanner with 32-channel radio-frequency coil at the Radiology Department in the university clinic of the LMU in Munich, Germany (Supplemental Information, Section 1.5). Both structural MRI (sMRI) and resting-state fMRI (rsfMRI) were acquired from all participants. T1 sMRI images were preprocessed using CAT12 (Supplementary Information, Section 1.6). rsfMRI preprocessing was divided into two main processes: core steps included realignment, coregistration, warping to Montreal Neurological Imaging (MNI) space and smoothing, whereas denoising steps comprised of motion correction using time series despiking with the BrainWavelet Toolbox (http://www.brainwavelet.org/) [52], background filtering and temporal band-pass filtering (0.01–0.08 Hz), extracting signal from white matter (WM) and cerebrospinal fluid (CSF), correcting for movement (Friston 24 movement parameters) [53] and calculating framewise displacement (FD) for each subject to determine inclusion [54] (Supplementary Information, Section 1.6).

Following sMRI and rsfMRI preprocessing, the brain was parcellated into 160 regions of interest (ROIs) according to the Dosenbach functional atlas [55]. We extracted the mean signal from 10 mm spheres centered at each ROI using the MarsBaR Toolbox [56] version 0.42. Next, the Pearson’s correlation of average time series between pairwise ROIs was calculated within Matlab R2015 using in-house scripts—resulting in 12720 rsFC for each participant. Connectivity matrices were generated for each subject in both the intervention sample and the original diagnostic classification sample.

### Machine learning strategy

**Fig. 1:**
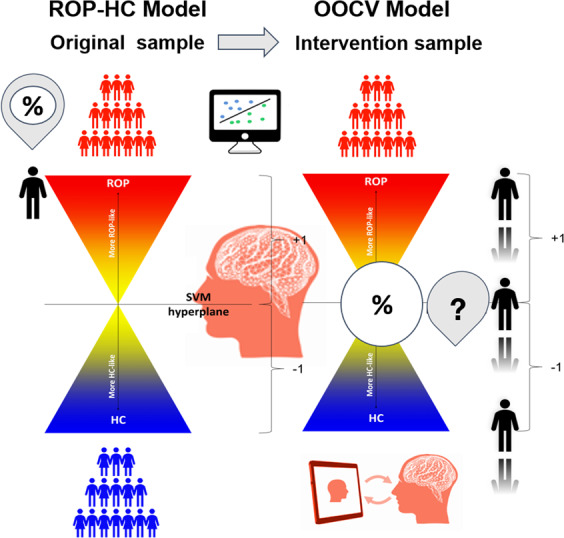
Proposed model depicting the application of a healthy-to-psychosis-like spectrum that could be used for monitoring treatment response to CCT. rsFC correlation matrices are entered into the SVM classification model to distinguish HC from ROP in an external sample. Using OOCV, the model is validated on patients who underwent the intervention sample at two time-points. Changes in decision scores are compared at the two time-points (FU-T0) in order to measure the direction of shift across the hyperplane based on rsFC.

The machine learning software NeuroMiner [39] version 1.0 was used to set up the machine learning analysis pipeline to extract multivariate decision rules from the rsFC data using an out-of-sample cross-validation (OOCV) strategy. First, a HC-ROP rsFC classifier was built to identify a disease-related rsFC signature. To investigate whether this disease-related signature could be used to track neural response to CCT in ROP patients, models generated for HC-ROP classification were applied to the intervention sample at both T0 and FU using OOCV. Here, we expected to identify a pattern of rsFC anomalies that not only classified HC and ROP with high accuracy, but that could also identify a set of individuals whose rsFC would shift to a more healthy-like rsFC pattern across the SVM hyperplane (Fig. 1).

### Machine learning analysis pipeline

NeuroMiner was used to create a predictive model that could separate patients with ROP from HC based on rsFC in the original diagnostic classification sample. To avoid overfitting, test the estimation of the model’s generalizability, and prevent information leakage between training and test participants, repeated-nested double cross-validation (CV) was employed [57, 58] (Supplementary Information, Section 1.7). This CV structure embeds a 10-fold inner CV cycle (CV1), where models are generated, in another super-ordinate 10-fold outer CV cycle (CV2), which is ultimately used to test the model’s generalizability [59, 60]. Both inner and outer CV cycles were permuted 10 iterations. Within CV1, matrices were pruned of zero-variance features, and sex and IQ effects were regressed out of the feature set using a partial correlation method. Then, a dimensionality reduction procedure was applied using Principal Component Analysis (PCA) in the CV1 training data to reduce the risk of overfitting and increase the generalizability of classification models [61] following previous methods [62]. Principal component (PC) scores were 0–1 scaled and fed to a linear class-weighted Support Vector Machine (SVM) algorithm (LIBSVM 3.1.2 L1-Loss SVC) [39, 63] to detect a set of PCs that optimally predicted the training and test cases’ labels in a given CV1 partition. The default regularization parameter of C = 1 was used within CV1 [64]. This analysis pipeline was subsequently applied to each k-fold and N-permutation CV2 cycle, determining the participant’s classification (HC vs. ROP) through majority voting. Statistical significance was assessed through permutation testing [57, 65], with α = 0.05 and 1000 permutations (Supplementary Information, Section 1.7).

### Validation analyses of classifier

The HC-ROP classifier built on the independent sample was subsequently applied to the intervention sample at T0 and FU without any in-between retraining using OOCV. The OOCV model provides a subject-specific linear SVM decision score at each timepoint for every ROP patient in the intervention sample. Positive decision scores indicate a predicted class membership of ROP, whereas negative decision scores indicate a predicted class membership belonging to HC. The difference in decision scores between the two time-points (FU-T0), that we address as psychosis-likeness change, provides an estimate of the direction of shift across the SVM hyperplane following CCT. Positive differences indicate a shift in the more psychosis-like direction, whereas negative differences indicate a shift in the more healthy-like direction across the SVM hyperplane. The measured changes in decision scores between the two time-points serve to verify if the multivariate rsFC signature from psychosis-like to healthy-like has been altered in the CCT intervention group. We performed platt scaling [66] to calibrate the decision score and assure that SVM predicted probabilities match the expected distribution of probabilities for each class. We calibrated the trained model by fitting the logistic regression to decision scores of the original HC-ROP model and applied this to the decision scores of the intervention data set. The HC-ROP classifier built on the LMU independent sample was additionally applied to three independent samples without any in-between retraining using OOCV in order to further assess generalizability of our model (Supplementary Information, Section 1.8, Table S5). We conducted additional correlational analyses to confirm our results are not biased by antipsychotic medication intake (Supplementary Information, Section 1.8, Table S6). We also ran additional correlational analyses to assess the associations between the psychosis-likeness model and 1) unhealthy consumption (e.g., cigarettes, alcohol), 2) variables indicative of socio-economic status (education and occupation of parents), patients functioning (GAF), traumatic experiences (Childhood trauma Questionnaire, CTQ [67, 68]) and age of illness onset (Supplementary Information, Section 1.8, Table S7).

### Statistical analyses of clinical and cognitive data

The following analyses were carried out in Jamovi version 1.1.9 (https://www.jamovi.org/), with a significance level of α = 0.05, with False Discovery Rate (FDR) correction for multiple comparisons [69]. Participants identified as outliers on cognitive domains (>2 SD) were excluded from further analyses. Demographic differences between groups were assessed using independent *t*-tests for continuous variables and chi-square tests for categorical variables. Repeated measures ANOVA was used to assess changes in cognition over time (1) based on SPC, (2) psychosis-likeness change, and (3) the interaction of SPC and psychosis-likeness change. Post-hoc analyses investigating the direction of effects were done using paired-samples t-tests. Effect sizes were reported using Cohen’s d [70].

## Results

### Group-level sociodemographic and clinical data

#### Independent sample (HC-ROP)

At baseline there were significantly more females in the HC group as compared to the patient group (*df* = 1, *χ*^*2*^ = 6.39, *P* = 0.012). Patients had significantly fewer years of education (*T*[86] *=* 2.51, *P* = 0.014), and lower premorbid IQ (*T*[89] = 2.80, *P* = 0.006) than HC individuals (Table 1). Patients with ROP showed significantly lower levels of functioning in all measures at T0 including GAF Disability and Impairment (*T*[88] = 26.91, *P* < 0.001), GF-R (*T*[88] = 12.24, *P* < 0.001), and GF-S (*T*[88] = 12.24, *P* < 0.001).

#### Intervention Sample (maintainers - improvers)

At baseline, there were no significant differences between maintainers and improvers in demographic characteristics, symptom severity, functioning, number of days between assessments, training intensity or antipsychotic medication (*P* > 0.05) (Table 2). The performance on all cognitive domains, except for verbal learning at baseline (*T*[24] = 2.18, *P* = 0.04) was balanced between the maintainers and improvers. We observed a marginally significant between groups effect on social cognition FU scores (*F*[1,25] = 4.45, *P* = 0.046), while controlling for T0 performance (*F*[1,25] = 4.08, *P* = 0.055). Although symptoms and functioning improved over time in all measures, there were no differences based on SPC (Table S3).

#### Resting-state functional connectivity prediction performance

**Fig. 2:**
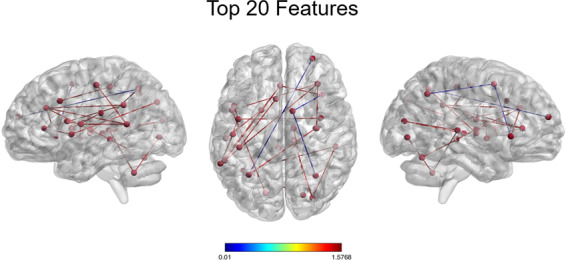
Depiction of the cross-validation ratio-based most reliable connections driving the classification between HC and ROP. The inter- and intrahemispheric connectivities of the top 20 features were extracted using a percentile rank of ~99.99% mapped onto the brain using BrainNet Viewer. Details of the regions that comprise the top 20 features are depicted in Table S8 in the Supplement. Blue lines indicate higher connectivity degree in the HC group; red lines indicate greater connectivity in the ROP group. Reliability is defined as the mean value of the SVM weight divided by its standard error across all the generated models in the cross-validation scheme.

The HC-ROP classifier correctly discriminated patients with ROP from HC with a cross-validated balanced accuracy (BAC) of 65.54% (sensitivity = 54.29%, specificity = 76.79%) and was significant (*P* = 0.01). Detailed statistics of the classification model are reported in Table S4. Inspection of the mean feature weights generated within the CV framework revealed that the rsFC connections driving correct classification between ROP and HC were long-range connections between (1) left parietal and right frontal lobe and (2) bilateral parietal lobe and thalamus, and short-range connections between (1) left parietal and left occipital area (2) right temporal and right angular gyrus, (3) left inferior temporal with right insula and left cerebellum, and (4) bilateral temporal lobe with bilateral thalamus (Fig. 2, Table S7). The connectivity patterns were mainly characterized by stronger FC associations in patients as compared to HC (Fig. 2) whereas only a few fronto-parietal and temporal-insular connectivities showed stronger connectivity in HC as compared to ROP patients (Fig. 2).

**Fig. 3:**
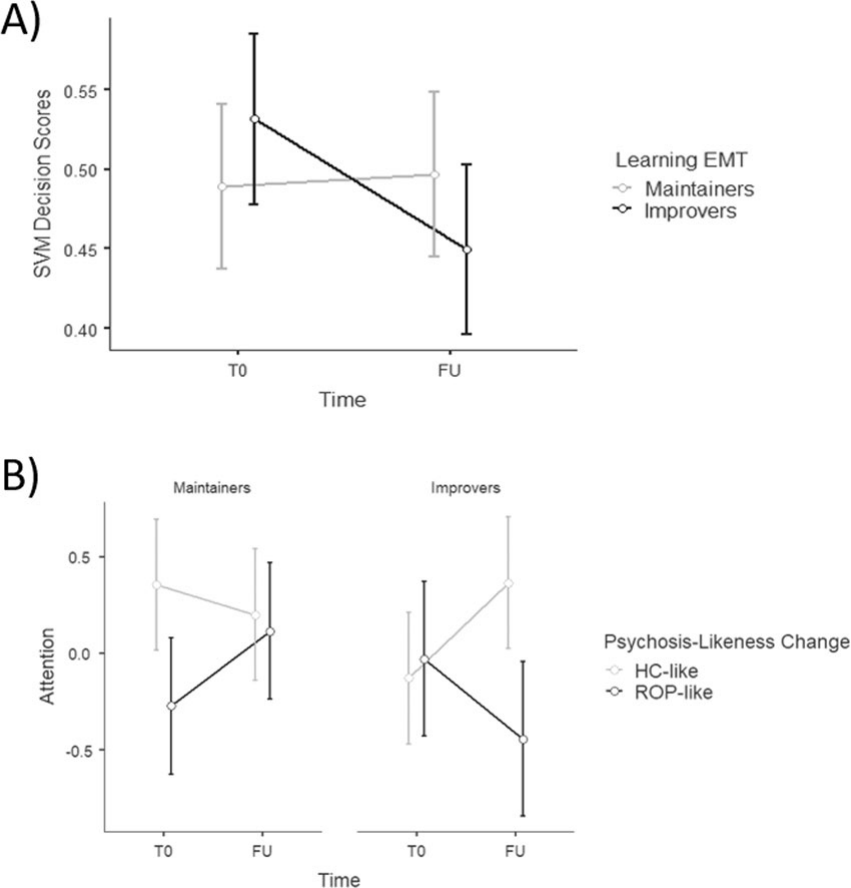
Decision scores and cognitive changes following computerized cognitive training. **a** SVM decision score change, reflecting the degree of psychosis-likeness based on resting-state functional connectivity (rsFC), in maintainers versus improvers and **b** attentional change based on shift across the hyperplane using rsFC and sensory processing change. Higher SVM decision scores reflect more psychosis-like rsFC. Error bars represent standard error. EMT Emotion Matching Task, FU follow-up, HC healthy control, ROP recent onset psychosis, SVM Support Vector Machine, T0 baseline.

Applying the ROP-HC model generated within the independent PRONIA sample to the intervention sample resulted in a model sensitivity of 65.38% at baseline and 57.69% at follow-up. When looking across all patients in the maintainer and improver subgroups, rsFC patterns shifted in the healthy-like direction (i.e., a decrease in decision scores from T0 to FU), with no significant differences in the number of patients whose rsFC shifted in the healthy-like direction (maintainers = 8, improvers = 8) as opposed to the psychosis-like direction (maintainers = 6, improvers = 4; *df* = 1, *χ*^*2*^ = 0.25, *P* = 0.62). Although there were no significant differences between maintainers and improvers in psychosis-likeness changes over time (*F*[1,25] = 0.96, *P* = 0.34), the overall shift to the healthy-like decision scores seems to be driven by a shift to the healthy-like part of SVM hyperplane in improvers (ES[Cohen’s d] = −0.35), whereas maintainers showed rather stable decision score values from T0 to FU (ES[Cohen’s d] = 0.03; Fig. 3a; Supplementary Information, Fig. S3 [A-B]).

Comparing maintainers and improvers further, we found a significant interaction between the group and the change in decision scores on the attentional gain (*F*[1,23] = 8.13, *P* = 0.01, [*P* = 0.06 with FDR correction]; Fig. 3b; Supplementary Information, Fig. S3 [C-D]). However, the effect of the group (*F*[1,23] = 0.06, *P* = 0.81) and decision score change (*F*[1,23] = 0.13, *P* = 0.72) alone on the attentional change was not significant. We observed a moderate effect size of improvement in attention despite psychosis-likeness change in the psychosis-like direction on the SVM hyperplane only in patients who showed intact SPC at baseline and maintained peak performance throughout the CCT (*T*[13] = 1.26, *P* = 0.26, ES = 0.51). Contrarily, attentional gains showed a large effect size in the ROP patients who showed impaired SPC at baseline only if the rsFC shifted to the healthy-like side of the SVM hyperplane (*T*[11] = 2.29, *P* = 0.06, ES = 0.87).

## Discussion

In this study, we performed a proof-of-concept analysis aimed at investigating the potential utility of rsFC to assess and monitor individual neural response to CCT. This is, to the best of our knowledge, the first study utilizing a machine learning rsFC model to investigate change of psychosis-likeness in response to CCT and associate it to changes in cognition and sensory processing.

To achieve this, we employed a model that was built on an independent sample of LMU ROP patients not undergoing the intervention, providing us with a quantifiable clinical outcome measure of psychosis-likeness change across the HC-ROP continuum with a BAC of 65.54%. This BAC is within the range of classification accuracies that utilize the resting-state modality for classifying chronic and first-episode psychosis patients from healthy controls [71].

After showing a solid generalizability of this model to the CCT sample, we followed the notion that various types of sensory [19] and multimodal plasticity impairments [72] may be differentially susceptible to interventions [37]. We used EMT as a proxy for sensory processing and created two patient groups based on the median split of SPC. We identified a subgroup of ‘improvers’ who initially presented with sensory processing impairments, however showed significant improvements in SPC throughout the course of the CCT. The other subgroup of ‘maintainers’ initially presented with unimpaired sensory processing and maintained peak performance throughout CCT at the optimal psychophysical level. We found that rsFC psychosis-likeness change in these two subgroups was differentially associated with attentional gains in response to CCT. Although we did not find a significant difference between improvers and maintainers in psychosis-likeness changes over time, the improvers showed a stronger change in psychosis-likeness to the healthy rsFC pattern. Importantly, these rsFC shifts seemed to be accompanied by attentional gains in improvers, while psychosis-likeness change in maintainers appeared compensated by efficient sensory processing that helped this subgroup nevertheless achieve attentional gains. Improvements in the attention domain after 10 h training is consistent with previous findings that improvements in low-order cognitive functions via drill-and-practice techniques precede gains in higher-order cognitive domains [73].

Stepping back to understand the resting-state pattern underlying psychosis-likeness in our original HC-ROP model, we observed widespread changes in both cortical and subcortical functional connectivities. We observed reduced rsFC between fronto-parietal regions and thalamo-cortical areas which successfully distinguished ROP patients from HC group, that may indicate less disturbed neuroplasticity in areas of top-down regulatory control, highly relevant for attentionally demanding cognitive tasks.

The importance of preserved fronto-parietal [13] and thalamo-cortical connectivity [66] is critical for normal cognitive functioning, in particular attention and sequential planning [74, 75], and relevant for mechanisms of learning in CCT. Our findings support this notion as the improvers, whose psychosis-likeness decreased or remained healthy-like, were able to translate cognitive skills acquired during CCT to attentional gains. Conversely, maintainers showed greater transfer effects to the domain of attention despite preserved psychosis-like rsFC, possibly due to their efficient sensory processing at baseline that served as cognitive reserve [14]. Our results suggest that improvement in attention may depend on an association between more healthy-like whole-brain rsFC patterns and efficient sensory processing during CCT and demonstrates feasibility of using resting-state as a valid biomarker. In line with our work, a recent fMRI study using resting-state connectivity networks was able to predict medication‐class of response in hard-to-diagnose patients [76], further supporting the utility of resting-state fMRI in the ‘real-world’ clinical context. In the recent meta-analysis on the utility of resting-state as biomarker, the authors warn about its moderate test-retest variability, while at the same time highlighting the complexity of its application and circumstances that improve the reliability of this neuroimaging modality [40, 77]. Future studies are necessary to determine the exact methodological conditions necessary to optimize the utility of neuroimaging to reliably trace the response to pharmacological and non-pharmacological interventions.

Several limitations of the present study need to be considered. First, the current study used a relatively short CCT as we wanted to keep the intervention duration comparable to the duration of clinical treatment. Our intention was to provide greater resemblance to the real-world clinical setting that appears common in many other health centers across Europe [78], and provides a strong clinical care framework due to the initial stay of the patients at the ward or frequent clinical checks. However, we cannot claim that ROP patients who did not respond with an improvement of rsFC pattern and did not show efficient SPC learning would not achieve neural ‘recovery’ associated with enhancement of cognition with a slightly different form of intervention, longer duration, or implementing more diverse protocols [7]. Second, we attempted to operationalize sensory processing during CCT by using a median split to categorize patients into improvers and maintainers. However, our approach may limit the generalizability of our findings and needs to be further investigated in future studies. Third, while the CCT in this study uses social stimuli, we have not observed any interaction between psychosis-likeness change and social cognition. While we measured performance on facial affect recognition, which represents only one domain of social cognition, a greater number of social cognitive measures would be needed to capture social cognition improvement at a fine-grained level [79]. Fourth, though we were not able to assess long-term effects of the intervention in an additional follow-up session, investigating durability effects of the intervention would be crucial for future studies. Finally, though we followed the generalizability rule in MVPA, including an independent sample in the study to generate the model and tested the generalizability of this model to three additional independent samples across multiple sites, future studies replicating our findings in multi-site cohorts with larger numbers of participants are warranted.

Prospectively, this MVPA approach may be integrated into individual early identification and intervention programs, thus resulting in a likely cheaper and more effective personalized psychiatry application [80, 81]. Psychotic disorders are highly heterogeneous at many levels, from biological pathways to clinical presentation and usage of the neuromonitoring approach may lead to faster identification of individuals with shared biological pathways that show a greater potential to improve through CCT [82].

## Funding and disclosure

This study was supported by the National Institute of Mental Health under Award Numbers R43 1 R43 MH121209-01 (PI:BB), EU-FP7 project PRONIA (“Personalised Prognostic Tools for Early Psychosis Management”) under the Grant Agreement No° 602152 (PI: NK) and NARSAD Young Investigator Award of the Brain & Behavior Research Foundation No° 28474 (PI: LK-I).

BB is Senior Scientist at Posit Science, a company that produces cognitive training and assessment software. The training programs described in this study were provided for research purposes free of charge by Posit Science. All other authors report no conflict of interest. RU reports grants from Medical Research Council, grants from the National Institute for Health Research, and personal fees from Sunovion, outside the submitted work. NK, JK, and RS received honoraria for talks presented at education meetings organized by Otsuka/Lundbeck. All other authors report no biomedical financial interests or potential conflicts of interest. Open Access funding enabled and organized by Projekt DEAL.

## Supplementary information

Supplementary Information
