# Supplementary material for: A multivariate neuromonitoring approach to neuroplasticity-based computerized cognitive training in recent onset psychosis
Source: Neuropsychopharmacology. 2020 Oct 7;46(4):828–35. doi: 10.1038/s41386-020-00877-4 (PMC8027389; doi:10.1038/s41386-020-00877-4)
Supplement: Supplementary file 1 — Supplementary Information [file 41386_2020_877_MOESM1_ESM.doc]

**Supplementary Material**

**A Multivariate Neuromonitoring Approach to Neuroplasticity-Based Computerized Cognitive Training in Recent Onset Psychosis**

Shalaila S. Haas, PhD^1^, Linda A. Antonucci, PhD^2,3^, Julian Wenzel, MSc^4^, Anne Ruef, PhD^2^, Bruno Biagianti, PhD^5,6^, Marco Paolini, MD^7^, Boris-Stephan Rauchmann, MD^2,7^, Johanna Weiske, MSc^2^, Joseph Kambeitz^4^, MD, Stefan Borgwardt, MD^8^, Paolo Brambilla, MD^9,10^, Eva Meisenzahl, MD^11^, Raimo K.R. Salokangas, MD^12^, Rachel Upthegrove, MBBS FRCPsych, PhD^13,14^, Stephen J. Wood, PhD^13,15,16^, Nikolaos Koutsouleris, MD^*2^, Lana Kambeitz-Ilankovic, PhD^*2,4^

^1^ Department of Psychiatry, Icahn School of Medicine at Mount Sinai, New York, New York

^2^ Department of Psychiatry and Psychotherapy, Ludwig-Maximilian University, Munich, Germany

^3^ Department of Education, Psychology, Communication – University of Bari “Aldo

Moro”, Bari, Italy

^4^ University of Cologne, Faculty of Medicine and University Hospital of Cologne

^5^ Department of R&D, Posit Science Corporation, San Francisco, CA, USA

^6^ Department of Pathophysiology and Transplantation, Faculty of Medicine and Surgery, University of Milan, Italy

^7^ Department of Radiology, University Hospital, Ludwig-Maximilian University, Munich, Germany

^8^ Translational Psychiatry Unit (TPU), Department of Psychiatry and Psychotherapy, University of Luebeck, Germany

^9^ Department of Neuroscience and Mental Health, Fondazione IRCCS Ca' Granda Ospedale Maggiore Policlinico, Milan, Italy

^10^ Department of Pathophysiology and Mental Health, University of Milan, Milan, Italy

^11^ Department of Psychiatry and Psychotherapy, Medical Faculty, Heinrich-Heine University, Düsseldorf, Germany

^12^ Department of Psychiatry, University of Turku, Turku, Finland

^13^ School of Psychology, University of Birmingham, United Kingdom

^14^ Institute of Mental Health, University of Birmingham, Birmingham, United Kingdom

^15^ Orygen, the National Centre of Excellence for Youth Mental Health, Melbourne, Australia

^16^ Centre for Youth Mental Health, University of Melbourne, Melbourne, Australia

*Denotes equal contributions of authors


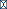
**corresponding autho**r**:**

Lana Kambeitz-Ilankovic, PhD

Department of Psychiatry and Psychotherapy, Faculty of Medicine and University Hospital of Cologne, Germany

Kerpenerstr.62, D- 50937 Cologne, Germany

e-mail: lana.kambeitz-ilankovic@uk-koeln.de

**Section 1.1: Sample Details**

**Inclusion and exclusion Criteria**

General inclusion criteria of the PRONIA study were age between 15 and 40 years, sufficient language skills for participation as well as capacity to provide informed consent/assent. General exclusion criteria were an IQ below 70, current or past head trauma with loss of consciousness (> 5 minutes), current or past known neurological or somatic disorders potentially affecting the structure or functioning of the brain, current or past alcohol dependence, or polysubstance dependence within the past six months, and any medical indication against MRI. In addition, HC exclusion criteria were: (1) any current or past DSM-IV axis disorder; (2) a positive familial history (1st degree relatives) for affective or non-affective psychoses; and (3) an intake of psychotropic medications or drugs more than 5 times/year and in the month before study inclusion. Specific ROP exclusion criteria included: (1) onset of psychosis exceeding the past 24 months (2) antipsychotic medication exceeding 90 days (cumulative in the past 24 months) with daily dose rate at or above minimum dosage of the “First Episode Psychosis” range of German Society for Psychiatry, Psychotherapy, and Nervous Diseases (DGPPN) S2 guidelines (equivalent to 5mg Olanzapine). For the ROP patients that underwent CCT, additional exclusion criteria included any prior cognitive training within the past 3 years.

**Intervention sample**

Patients were recruited into the study from June 2016 to December 2018. Of the 63 patients included in the study, 33 were randomly assigned by research assistants to an active 10-hour social CCT over the course of 5 weeks (30 minutes per session, 4-5 days per week)^1^ (Figure S1) using the research randomizer (https://www.randomizer.org/). For the randomization, we used 1 set with 60 numbers, ranging from 1-2 for each condition, with unique numbers set to “no”. Of the 33 patients who received CCT, 5 dropped out during the training period, one was excluded due to incompatible MRI parameters, and one due to having greater than 3 cognitive tests in which the participant was an outlier, leaving a total sample of 26 ROP patients who completed the intervention and were included in the subsequent analyses. As we were interested in gauging response to the intervention itself, in this study, we focused on patients that underwent the intervention.

A statistical power analysis was performed for sample size estimation, based on data from a comparable published study utilizing SCT^2^. The effect size in this study (d = 0.74), was considered to be medium-to-large using Cohen’s criteria^3^. With an α= 0.05 and power = 0.80, the projected sample size needed with this effect size (G * Power Version 3.14) is N = 24 per condition for the simplest between group comparison. Thus, our sample size of 26 participants was considered adequate for this study.

**Section 1.2: Computerized cognitive training (CCT) paradigm**

The first three training sessions took place at the Department of Psychiatry and Psychotherapy of the LMU. After three sessions, participants attended group training sessions in the clinic (N = 15) or trained from home (N = 11). The exercises described in Table S1 are meant to improve accuracy and speed of neural functions associated with processing social information^1^. The training program is structured in blocks with early blocks using stimuli meant to strengthen and refine responses, in order to accurately encode the parametric details of basic inputs and actions at high speed. Once these fundamental processes are consolidated, subsequent blocks utilize naturalistic properties to ensure that training results in progressive improvements in processing stimuli that apply to real-world performance.

Each block consists of 20-50 adaptive trials. Within each block, sophisticated adaptive tracking methods are employed to continuously adjust a single adaptive dimension of the task to capabilities of the participant. This adaptive process is based on a statistically optimal Bayesian approach that allows the exercise to rapidly adapt to an individual’s performance level, and maintain the difficulty of the stimulus sets at an optimal level for driving efficient learning. As the user progresses through the different blocks of each exercise, difficulty levels increase in: 1) stimulus complexity; 2) number of response alternatives; 3) stimulus and response presentation times. This ensures that the difficulty levels adjust to the appropriate levels based on a specific individual’s rate of learning. Block completion is based on user performance: once exercise-specific algorithms detect no additional improvements, the block terminates, and users are presented with an easier or harder block of the same exercise, depending on their performance.

Two metrics are available for each exercise: (1) baseline performance – the score reached the first time a participant completed any given exercise; (2) best performance – this is the best score reached in a training exercise at any point throughout the intervention. Correct trials are rewarded with auditory feedback, points and animations. Compliance is monitored by electronic data upload. Further details regarding the training are described by Nahum et al (2014)^1^.

**Section 1.3: Target engagement**

While all four exercises target early social sensory processing, we chose to study the Emotion Matching Task (EMT) as a potential proxy for target engagement, given its ability to capture the processing of basic social information. In this speeded exercise, participants are shown a target face displaying an emotion and then asked to select from a set of other images which one displays the same facial expression. The exercise is designed to improve the ability to make implicit speeded decisions about facial emotions.

Twenty-four unique blocks of EMT are available throughout the training. A decrease in presentation times indicates a succession of correct answers. The learning score for person (P) at level (L) is defined as the difference between the best score BP,L (i.e. lowest presentation time), and the baseline score IP,L (i.e. the initial score the level was played), divided by the standard deviation of baseline scores SDIL across all participants. Figure S2 depicts A) the learning score distribution plots for the two groups (maintainers and improvers) based on the median split and B) group differences between improvers and maintainers (*T* = -3.38, *p*=0.002).

**Section 1.4: Neurocognitive Assessment**

All patients recruited underwent a neurocognitive assessment which comprised of 9 tests: 1) Diagnostic Analysis of Non-Verbal Accuracy-2 (DANVA-2)^4^, 2) Wechsler Memory Scale, 3rd ed., spatial span subtest (forward and backward)^5^, 3) semantic and phonetic verbal fluency^6^, 4) Rey Auditory Verbal Learning Test ^7,8^, 5) Trail-Making Test (TMT) Part A and B^9^, 6) Continuous Performance Task - Identical Pairs (CPT-IP)^10^, 7) Self-Ordered Pointing Task (SOPT)^11^, 8) Wechsler Adult Intelligence Scale, 3rd ed., digit symbol coding task^5^, and 9) the Wechsler Adult Intelligence Scale, 3rd ed., Matrices and Vocabulary^5^ as a measure of premorbid intelligence. These tests were combined into specific domains^12^ by z score transforming the tests. When a domain consisted of multiple tests, the transformed scores were averaged. Global cognition was calculated by taking the aggregate mean across the 5 separate cognitive domains. Outliers (>2 SD) were excluded from further analyses.

**Section 1.5: Neuroimaging Acquisition**

T1 reference images were obtained using a multi-echo MPRAGE sequence with the following parameters: repetition time (TR) = 9.5 ms, echo time (TE) = 5.5 ms, flip angle = 8°, field of view = 250 x 250 mm, matrix size = 256 x 256; 190 contiguous sagittal slices of 1.0 mm thickness and a 1.0 mm gap, voxel size = .97 mm x .97 mm x 1 mm, pixel band width = 650 Hz. Blood Oxygenation Level Dependent (BOLD) images of the whole brain using an echo planar imaging (EPI) sequence were acquired in 53 ascending slices (TR = 3000 ms, TE = 30 ms, flip angle = 90°, field of view = 230 x 230 mm, 3.0 mm thickness and 3.0 mm gap, matrix size = 80x80, voxel size = 2.875 mm x 2.875 mm x 3 mm) using the intercommissural line (AC-PC) as a reference. rsfMRI scans resulted in 603 s duration (200 volumes) and subjects were instructed to keep their eyes open during the scan.

**Section 1.6: Neuroimaging Preprocessing**

The manual of the CAT12 toolbox (<http://www.neuro.uni-jena.de/cat12/CAT12-Manual.pdf>) details the processing steps applied to the structural images, consisting of (1) the 1st denoising step based on Spatially Adaptive Non-Local Means (SANLM) filtering^13^; (2) an Adaptive Maximum A Posteriori (AMAP) segmentation technique, which models local variations of intensity distributions as slowly varying spatial functions and thus achieves a homogeneous segmentation across cortical and subcortical structures^14^; (3) the 2nd denoising step using Markov Random Field approach which incorporates spatial prior information of adjacent voxels into the segmentation estimation generated by AMAP^14^; (4) a Local Adaptive Segmentation (LAS) step, which adjusts the images for white matter (WM) inhomogeneities and varying gray matter (GM) intensities caused by differing iron content in e.g. cortical and subcortical structures. The LAS step is carried out before the final AMAP segmentation; (5) a partial volume segmentation algorithm that is capable of modeling tissues with intensities between GM and WM, as well as GM and cerebrospinal fluid (CSF) and is applied to the AMAP-generated tissue segments; (6) a high-dimensional DARTEL registration of the image to a MNI-template generated from the MRI data of 555 healthy controls in the IXI database ([http://www.braindevelopment.org](http://www.braindevelopment.org/)). The registered GM images were multiplied with the Jacobian determinants obtained during registration to produce GM volume (GMV) maps.

rsfMRI preprocessing was divided into two main processes: core and denoising steps based on Patel et al.^15^. Core preprocessing consisted of the following and were performed using Statistical Parametric Mapping, version 12 (SPM12) (<https://www.fil.ion.ucl.ac.uk/spm/software/spm12/>) version 6685. After initially discarding the first 8 volumes, the remaining 192 images were slice-time corrected, and then unwarped and realigned to the first volume for head-motion correction. The time course of head motion was obtained by estimating the translations in each direction and the rotations in angular motion about each axis for each volume. Next, framewise displacement (FD) was calculated for each subject^16^. FD for the first volume of a run is by convention zero. Subjects with greater than 38.5 % of volumes with mean FD of > 0.50 mm were excluded from further analyses^17^.

Affine coregistration of images to structural images followed and were then resliced using 4th-degree B-Spline interpolation. The standard CAT12 template was converted from DARTEL space to MNI space using SPM12’s population to International Consortium for Brain Mapping 152 registration procedure. The resulting image was used as a deformation field to normalize all coregistered images to MNI space. Next, gray matter, white matter, and CSF masks were created using an image calculator procedure within SPM12 using thresholds of 0.20, 0.20, and 0.50 respectively. Subsequently, Friston 24 motion parameters^18^ including 6 motion parameters, 6 temporal derivatives, 6 quadratic terms and 6 quadratic expressions of the derivatives of motion estimates, were derived. Then mean individual signal estimates with variance regressed out from white matter and CSF were generated. Finally, functional volumes were masked using the gray matter mask to limit space and spatial smoothing using a Gaussian kernel of 6 mm full width at half-maximum was applied.

Denoising methods consisted of: motion correction using time series despiking (Wavelet Despike) with the BrainWavelet Toolbox (<http://www.brainwavelet.org/>)^15^. The following steps were done using the Resting-State fMRI Data Analysis Toolkit (REST version 1.8; <http://www.restfmri.net/>)^19^. Confound signal regression of the Friston 24 motion parameters, and residuals of white matter and CSF were applied. Finally, the images underwent background filtering and temporal band-pass filtering (0.01 - 0.08 Hz) was performed to reduce the effects of low-frequency drift and high-frequency noise^19^.

**Section 1.7 Multivariate Preprocessing Pipeline**

To allow for unbiased estimation of the model’s generalizability and prevent information leakage between subjects used for training the models and subjects used for validating decisions^20^, we built a double cycle, repeated nested cross-validation (CV) framework^21^. Nested CV implies the presence of an inner CV cycle embedded in another, super-ordinate, outer CV cycle which is ultimately used to test for models’ generalizability^22^. Both in inner and outer CV levels, we employed a 10-fold CV cycle. We extended nested CV to repeated nested CV^23^ at the outer CV2 cycle by randomly permuting the participants within their groups (number of permutations = 10) and repeating the CV cycle for each of these permutations. The principal components with the highest eigenvalues that cumulatively explained 80% of the variance were selected and the single subject rsFC were projected into the reduced principal components space^24,25^. Principal Components (PCs) scores derived from functional connectivity matrices were 0-1 scaled and a linear class-weighted Support Vector Machine (SVM) (LIBSVM 3.1.2 L1-Loss SVC) was used to detect a set of PCs that optimally predicted the training and test cases’ labels in given CV1 partition^26^, while taking into account differences in class size of the two groups (HC vs ROP).

In each variable evaluation step, the SVM algorithm modeled independently linear relationships between features and classification label (HC vs. ROP). In the linear kernel space, the SVM optimized a hyperplane that maximized separability between most HC-like and most ROP-like subjects (i.e., the Support Vectors). Based on the trained hyperplane, the algorithm then predicted subjects’ classification of the inner CV cycle by projecting its data into the learned kernel space and measuring their geometric distance to the decision boundary. This resulted in a decision value and a predicted classification label per participant. The default regularization parameter of C = 1 was used within CV1, as recommended by Fan et al.^27^, in order to avoid overfitting of the model.

To assign statistical significance to the observed prediction performance of our prediction models, we employed permutation testing^28^. We performed 1000 random permutations of the outcome labels (HC-ROP). For each permutation, we retrained all linear SVM models in the pooled repeated nested double CV experiment using the respective feature subsets obtained from the observed-label analyses. For each permutation, we accumulated the predictions of the random models into a permuted ensemble prediction for each outer cycle subject. Thus, we built a null distribution of out-of-training classification performance (accuracy) for every prediction based on the classifier prediction. Finally, we calculated the significance of the observed out-of-training accuracy as the number of events where the permuted out-of-training accuracy was higher or equal to the observed accuracy divided by the number of permutations performed. The significance of the model was determined at α=0.05.

To better understand which variables might inform HC and ROP classes at the single-subject level, we extracted the percentage difference in connectivity between HC and ROP for the 99th percentile of reliable connections, following published procedures^24^. Reliability for each connection is defined in terms of a Cross-Validation Ratio (CVR = mean(w)/standard error(w))^29^. In this formula, w represents the normalized individual weights from SVM models generated in the repeated-nested double CV scheme. Normalization is performed using the Euclidean norm of w, defined as s = w/||w||2^29^. A positive CVR for each pairwise connection indicates greater connectivity for ROP compared to HC, while a negative CVR for each pairwise connection indicates a decrease in connectivity for ROP compared to HC. CVR for the 99th percentile of most reliable connections^24^ of the classifier discriminating between ROP and HC are reported in Figure 3A and depicted using BrainNet Viewer (<https://www.nitrc.org/projects/bnv/>)^30^ in Figure 3.

**Section 1.8 Additional Validation and Reliability Analyses**

We applied the HC-ROP classifier built on the independent sample to three additional samples without any in-between retraining using OOCV in order to further assess generalizability of our model. The model was first applied to a replication sample recruited from the same PRONIA site as the sample used to create the model and the intervention sample (Munich). We additionally applied the model to the two additional German-speaking PRONIA sites, to further assess the generalizability of our model (Basel and Cologne). Results of these analyses are shown in Table S5.

In order to exclude that medication dose did not bias the psychosis-likeness changes in the intervention sample, we ran Spearmans’ rho correlation analysis between chlorpromazine equivalents and SVM decision scores at baseline and follow-up. Furthermore, we ran a two-sample t-test (between-subjects effect: classified vs misclassified ROP; dependent variable: chlorpromazine equivalents); at both baseline and follow-up. Results show that the decision scores of the rsFC classifier did not correlate with medication dose and did not differ between individuals correctly classified as ROP vs misclassified as HC (Table S6).

In order to better understand which aspects of psychosis are captured by the SVM decision plane, we ran additional correlational analyses with participant functioning, childhood trauma, and parental socio-economic status. For parental socio-economic status, education was reflected by highest degree obtained ([1] University degree, [2] Graduation qualifying University degree, [3] Other Graduation, [4] Without Graduation), and occupation was reflected by the highest occupation position worked ([1] Managers, [2] Professionals, [3] Technicians and associate professionals, [4] Clerical support workers, [5] Service and sales workers, [6] Skilled agricultural, forestry and fishery workers, [7] Craft and related trades workers, [8] Plant and machine operators, and assemblers, [9] Elementary occupations). Moreover, to exclude that the psychosis-likeness model built on the independent sample did not simply reflect unhealthy lifestyle we performed additional correlational analyses between cigarette and alcohol consumption in the PRONIA original sample and SVM decision scores. GAF and CTQ scores showed significant correlations with decision values across all individuals included in the psychosis-likeness model (GAF: r = -0.46, p < 0.001; CTQ: r = 0.24, p = 0.02). Results showed that the decision scores of the rsFC classifier did not correlate with cigarettes (r = 0.06; p = 0.74) or alcohol (r = 0.09, p = 0.40) consumption (Table S7). Associations between decision values and other demographic or clinical variables were not significant (p>0.05, Table S7).

**Figures**


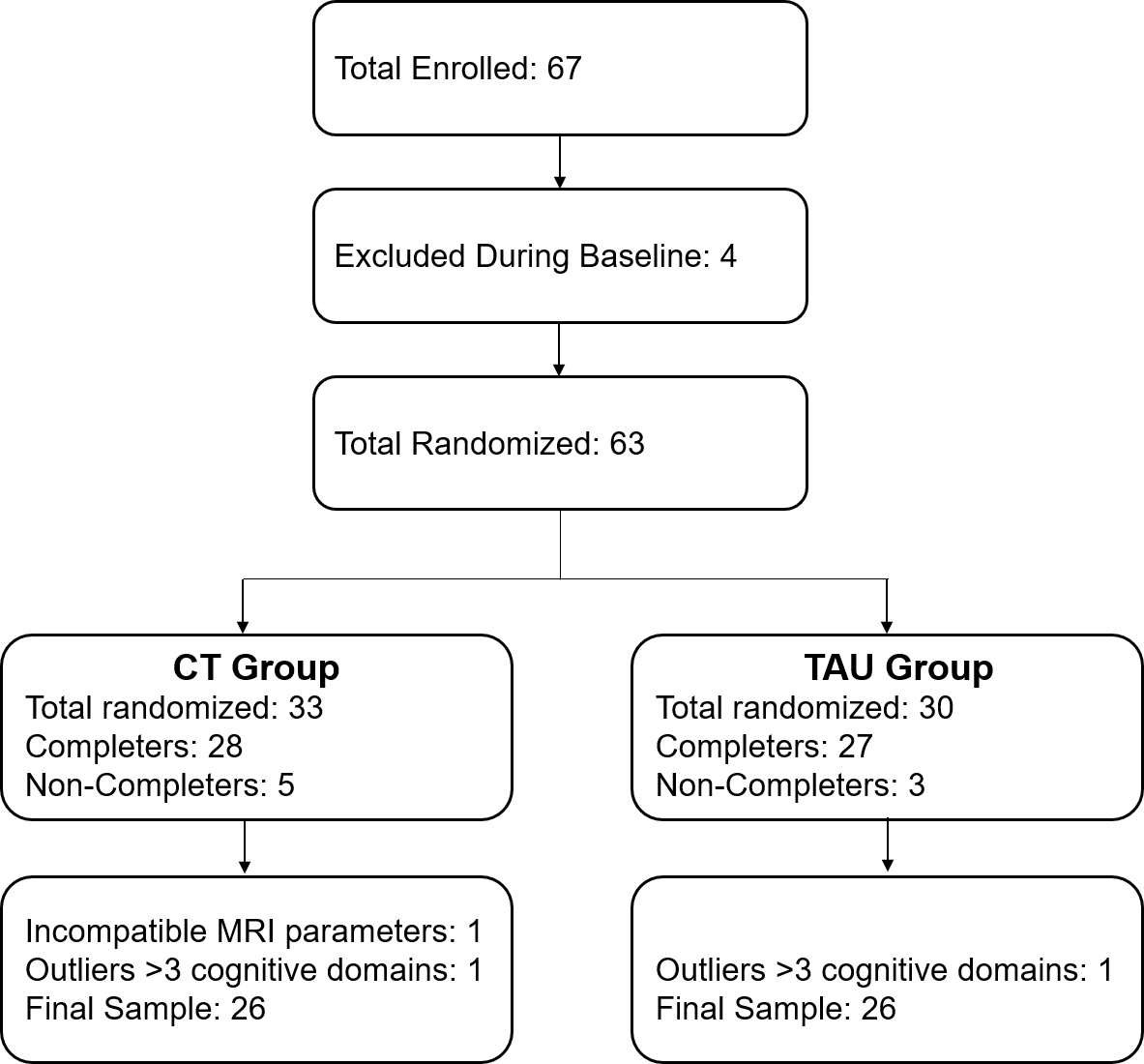


**Figure S1.** Flowchart of the study sample.


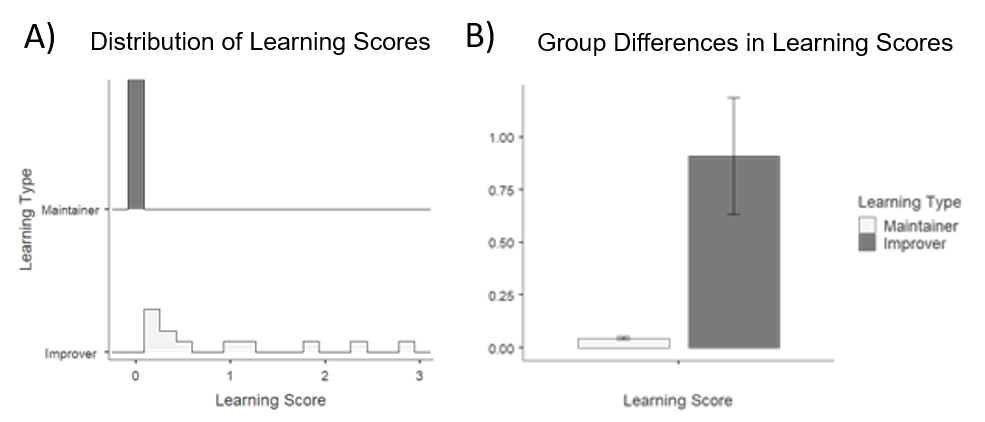


**Figure S2.** Graphs depicting A) the distributions of learning scores separated by learning type and B) group differences in learning scores in the intervention sample.


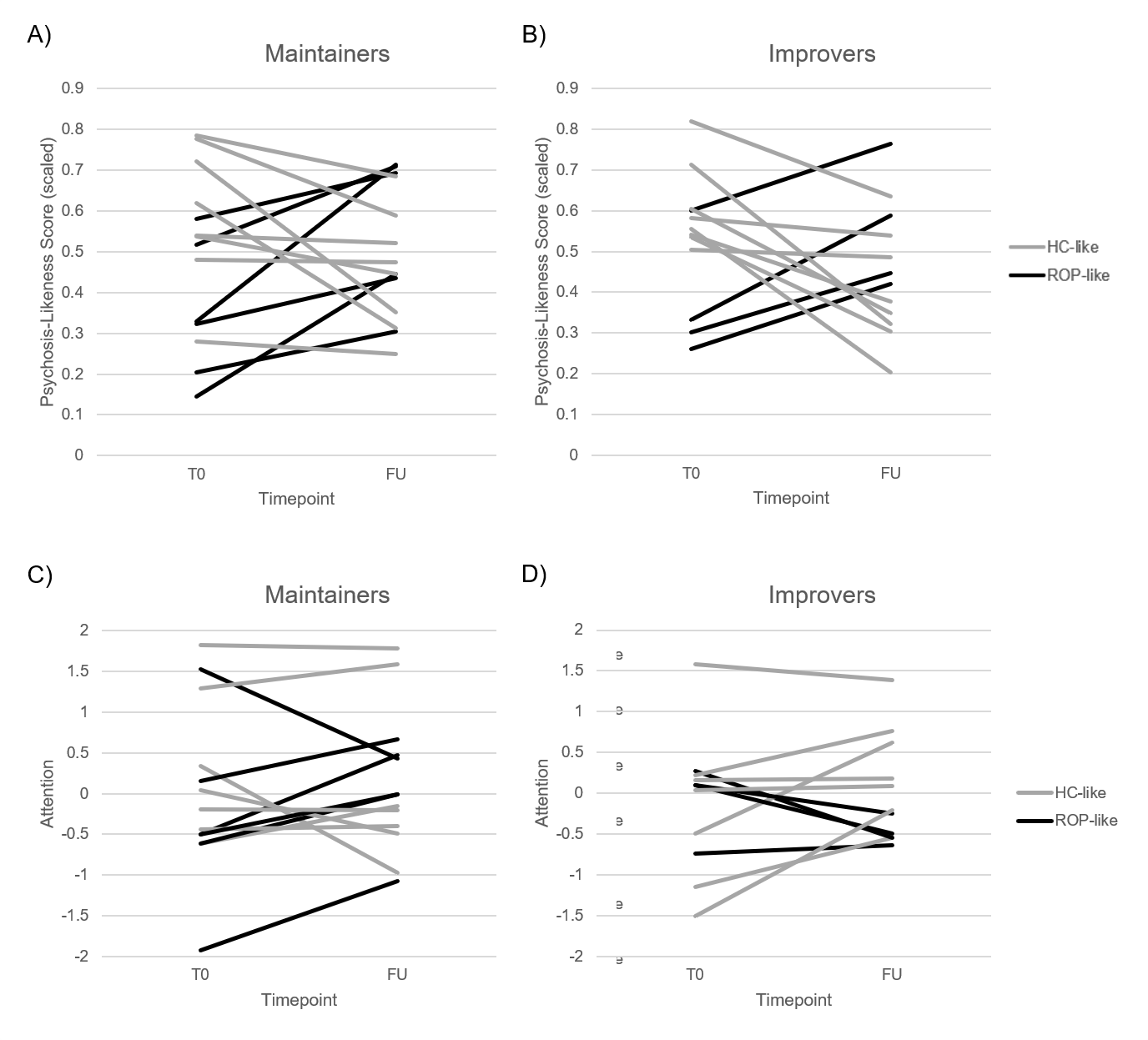


**Figure S3.** Spaghetti plots depicting the subject-specific changes in A) psychosis-likeness scores in maintainers and B) improvers and C) attention scores in maintainers and D) improvers grouped by learning type and B) group differences in learning scores in the intervention sample.

**Tables**

**Table S1.** Description of the social cognitive training exercises provided by Posit Science, Inc. (SocialVille) used for the intervention in the order of administration (1).

| Exercise | Trials per iteration^*^ | Description | Target |
| --- | --- | --- | --- |
| Speeded Face Matching | 20 | A speeded face matching task: Select the correct target face from an array of faces | Improve processing of facial features. |
| Speeded Facial Emotion Matching | 20 | A speeded facial emotion matching task: Select the face showing the same facial expression as the target face | Improve the ability to make implicit speeded decisions about facial emotion features. |
| Speeded Eye Gaze Matching | 40 | A speeded gaze matching task: Match gaze direction of target face | Improve processing speed for accurate identification of eye gaze direction. |
| Facial Emotion Continuous Performance Task (CPT) | 60 | A CPT task with facial expressions: Withhold response for neutral expressions (10 % of trials), respond quickly to emotional faces (90 %) | Improve the brain's ability to distinguish between emotionally expressive faces and neutral faces. |

^*^ Each exercise repeated several iterations of each exercise until approximately 7-8 minutes were complete.

**Table S2.** The seven cognitive domains assessed and a description of their respective tests.

| Neurocognitive domain | Cognitive test | Description of tests |
| --- | --- | --- |
| Social cognition | Diagnostic Analysis of Nonverbal Accuracy-2 | A test of social cognition measuring the ability to read nonverbal social information. |
| Speed of processing | Trail Making Test (TMT): Part A | A test of visual scanning and visuomotor tracking |
|  | Verbal Fluency: semantic | A verbal index of speed of processing |
|  | Wechsler Adult Intelligence Scale, 3rd ed., digit symbol coding task | A measure of visuomotor speed |
| Working memory | Wechsler Memory Scale, 3rd ed., spatial span subtest | A measure of nonverbal working memory |
| Verbal learning | Rey Auditory Verbal Learning Test (RAVLT) | A list of 15 words presented 5 times, which must be recalled from memory |
| Attention | Continuous Performance Task - Identical Pairs (CPT-IP) | A measure of attention and vigilance |
| Global cognition | Composite across all cognitive measures included above (average z-score) | A global measure of cognitive functioning |

**Table S3:** Scores on cognitive measures, symptom ratings, and functional outcomes at baseline and follow-up of participants with good versus poor learning profiles. ^1^Two subjects were excluded from analyses due to having attention scores greater than 2 standard deviations from the mean. EMT = Emotion Matching Task; FU = follow-up; GAF = Global Assessment of Functioning; PANSS = Positive and Negative Syndrome Scale; T0 = baseline.

|  | Maintainers EMT  (N = 14) | | Improvers EMT  (N = 12) | | Main Effect of Time  *F* (*P*) | Interaction (Group x Time)  *F* (*P*) | |
| --- | --- | --- | --- | --- | --- | --- | --- |
|  | T0  (SD) | FU  (SD) | T0  (SD) | FU  (SD) |
| **Cognition** |  |  |  |  |  |  | |
| Global cognition | 0.14 (0.55) | 0.19 (0.67) | 0.16 (0.71) | 0.22 (0.50) | 0.002  (0.96) | 0.39  (0.54) | |
| Social cognition | 0.10 (0.98) | 0.37  (0.75) | -0.12 (1.06) | -0.44 (1.11) | 0.01  (0.92) |  | 1.94  (0.18) |
| Speed of processing | 0.03 (0.83) | 0.08 (0.94) | -0.03 (0.77) | -0.10 (0.58) | 0.002  (0.97) |  | 0.32  (0.58) |
| Working memory | 0.04 (0.95) | 0.08 (0.97) | -0.04 (0.76) | -0.01 (0.73) | <0.001  (0.99) |  | 0.04  (0.84) |
| Verbal Learning | 0.37 (0.72) | 0.27 (1.09) | -0.43 (1.14) | -0.31 (0.82) | 0.003  (0.96) |  | 0.53  (0.47) |
| Attention^1^ | 0.03  (1.03) | 0.12 (0.86) | -0.13 (0.83) | 0.03 (0.65) | 0.37  (0.55) |  | 8.13  (0.01) |
| **Functional Outcome** |  |  |  |  |  |  | |
| GAF global rating past month | 46.25 (13.86) | 58.82 (13.54) | 48.00 (16.87) | 57.83 (8.92) | 11.42 (0.002)** | 0.17  (0.68) | |
| Global Functioning - Role | 4.57 (1.45) | 5.93 (1.49) | 4.25 (1.55) | 5.08 (1.38) | 14.62  (<0.001)*** | 0.84  (0.37) | |
| Global Functioning - Social | 6.00 (1.30) | 6.71 (1.27) | 6.00 (0.95) | 6.42 (1.00) | 7.14  (0.013)* | 0.50  (0.49) | |
| **Symptoms** |  |  |  |  |  |  | |
| PANSS total | 66.07 (15.61) | 43.79 (16.72) | 69.83 (17.94) | 44.42 (12.75) | 30.98 (<0.001)*** | 0.13  (0.72) | |
| PANSS positive | 19.21 (6.12) | 10.29 (4.25) | 19.83 (5.88) | 10.50 (3.12) | 48.26 (<0.001)*** | 0.02  (0.879) | |
| PANSS negative | 13.43 (5.24) | 10.29 (4.91) | 15.83 (6.19) | 11.33 (4.25) | 9.31 (0.005)** | 0.29  (0.59) | |
| PANSS general psychopathology | 33.43 (9.10) | 23.21 (8.79) | 34.17 (9.11) | 22.58 (7.00) | 22.68 (<0.001)*** | 0.09  (0.77) | |

**Table S4.** Performance of the independent [HC](https://docs.google.com/document/d/1WDi0rOIy4m7AJmYG1de8qImD2xNSI-62/edit#heading=h.1t3h5sf)-[ROP](https://docs.google.com/document/d/1WDi0rOIy4m7AJmYG1de8qImD2xNSI-62/edit#heading=h.3dy6vkm) classification model based on [rsFC](https://docs.google.com/document/d/1WDi0rOIy4m7AJmYG1de8qImD2xNSI-62/edit#heading=h.4d34og8) and validation performance in the study sample at [T](https://docs.google.com/document/d/1WDi0rOIy4m7AJmYG1de8qImD2xNSI-62/edit#heading=h.3rdcrjn)0 and [FU](https://docs.google.com/document/d/1WDi0rOIy4m7AJmYG1de8qImD2xNSI-62/edit#heading=h.26in1rg) without any in-between re-training.

|  | TP | TN | FP | FN | Sens [%] | Spec [%] | BAC [%] | FPR [%] | PPV [%] | NPV [%] | AUC |
| --- | --- | --- | --- | --- | --- | --- | --- | --- | --- | --- | --- |
| **Classification:** |  |  |  |  |  |  |  |  |  |  |  |
| HC versus ROP  **Out-of-sample validation:** | 19 | 43 | 13 | 16 | 54.29 | 76.79 | 65.54 | 23.21 | 59.38 | 72.88 | 0.76 |
| T0 | 18 | - | - | 8 | 69.23 | - | - | - | 100 | 0 | - |
| FU | 14 | - | - | 12 | 53.85 | - | - | - | 100 | 0 | - |

**Table S5.** Performance of the independent [HC](https://docs.google.com/document/d/1WDi0rOIy4m7AJmYG1de8qImD2xNSI-62/edit#heading=h.1t3h5sf)-[ROP](https://docs.google.com/document/d/1WDi0rOIy4m7AJmYG1de8qImD2xNSI-62/edit#heading=h.3dy6vkm) classification model based on [rsFC](https://docs.google.com/document/d/1WDi0rOIy4m7AJmYG1de8qImD2xNSI-62/edit#heading=h.4d34og8) and validation performance in the replication sample within the same site (Munich) and two additional PRONIA sites (Basel and Cologne) without any in-between re-training. Details pertaining to the site descriptions of PRONIA have been outlined in Koutsouleris et al., 2018.

|  | TP | TN | FP | FN | Sens [%] | Spec [%] | BAC [%] | FPR [%] | PPV [%] | NPV [%] | AUC |
| --- | --- | --- | --- | --- | --- | --- | --- | --- | --- | --- | --- |
| **Classification:** |  |  |  |  |  |  |  |  |  |  |  |
| HC versus ROP  **Out-of-sample validation:** | 19 | 43 | 13 | 16 | 54.29 | 76.79 | 65.54 | 23.21 | 59.38 | 72.88 | 0.76 |
| Munich Replication | 18 | 6 | 1 | 10 | 64.29 | 85.71 | 75.00 | 14.29 | 94.74 | 37.50 | 0.68 |
| Cologne Discovery | 11 | 46 | 10 | 16 | 40.74 | 82.14 | 61.44 | 17.86 | 52.38 | 74.19 | 0.70 |
| Basel Discovery | 10 | 27 | 10 | 11 | 47.62 | 72.97 | 60.30 | 27.03 | 50.00 | 71.05 | 0.71 |

**Table S6.** Association tests between medication dose and our rsFC classifiers’ decision scores performed at baseline (T0) and follow-up (FU) in the intervention sample.

| Association test between rsFC classifier’s decision scores and medication dose | Correlation  analysis,  Spearman’s  rho | Correlation  analysis,  p value | Two-sample  t-test,  T score | Two-sample  t-test,  p value |
| --- | --- | --- | --- | --- |
| Intervention sample baseline (N=26) | 0.01 | 0.95 | -0.12 | 0.91 |
| Intervention sample follow-up (N=26) | 0.27 | 0.2 | -0.78 | 0.45 |

**Table S7.** Association tests between various aspects of psychosis and our rsFC classifiers’ decision scores in the original sample.

| Association test between rsFC classifier’s decision scores and medication dose | Correlation  analysis,  Pearson’s  r (all) | Correlation  analysis,  p value (all) | Correlation  analysis,  Pearson’s  r (ROP) | Correlation  analysis,  p value (ROP) |
| --- | --- | --- | --- | --- |
| Global assessment of functioning | -0.46 | <0.001*** | -0.08 | 0.65 |
| Childhood Trauma Questionnaire Score | 0.24 | 0.02* | -0.12 | 0.51 |
| Cigarettes (average number/day) | 0.06 | 0.74 | 0.05 | 0.84 |
| Alcohol (units) | 0.10 | 0.40 | -0.06 | 0.77 |
| Age at illness onset | - | - | -0.25 | 0.15 |
| Mother’s highest-level education | -0.009 | 0.93 | 0.04 | 0.83 |
| Mother’s highest occupational level | 0.12 | 0.30 | 0.03 | 0.87 |
| Father’s highest-level education | 0.03 | 0.79 | 0.13 | 0.50 |
| Father’s highest occupational level | 0.03 | 0.75 | -0.05 | 0.80 |

Note: * p < .05, ** p < .01, *** p < .001

| **Table S8.** The top 20 features were extracted using a percentile rank of approximately 99.99%. | | | | | | | | | | | |
| --- | --- | --- | --- | --- | --- | --- | --- | --- | --- | --- | --- |
| Region 1 | Hemi | Coordinates  (x, y, z) | | | Region 2 | Hemi | Coordinates  (x, y, z) | | | Mean Weight | CV Ratio |
| **Occipital-Parietal** | | | | | | | | | | | |
| occipital | L | -16 | -76 | 33 | parietal | L | -24 | -30 | 64 | 0.79 | 10.82 |
| occipital | L | -16 | -76 | 33 | inferior parietal sulcus | L | -36 | -69 | 40 | 0.72 | 10.07 |
| occipital | L | -9 | -72 | 41 | parietal | L | -24 | -30 | 64 | 0.72 | 11.50 |
| occipital | L | -9 | -72 | 41 | parietal | L | -38 | -27 | 60 | 0.70 | 11.93 |
| **Temporal-Thalamus** | | | | | | | | | | | |
| temporal | L | -59 | -47 | 11 | thalamus | L | -12 | -12 | 6 | 0.77 | 10.87 |
| temporal | R | 59 | -13 | 8 | thalamus | R | 11 | -12 | 6 | 0.75 | 11.19 |
| temporal | L | -53 | -37 | 13 | thalamus | L | -12 | -12 | 6 | 0.75 | 10.70 |
| temporal | R | 51 | -30 | 5 | thalamus | R | 11 | -12 | 6 | 0.72 | 10.49 |
| temporal | L | -54 | -22 | 9 | thalamus | L | -12 | -12 | 6 | 0.72 | 11.41 |
| temporal | L | -53 | -37 | 13 | thalamus | R | 11 | -12 | 6 | 0.70 | 11.95 |
| **Temporal-Cerebellum** | | | | | | | | | | | |
| inferior temporal | L | -59 | -25 | -15 | inferior cerebellum | L | -6 | -79 | -33 | 0.74 | 8.81 |
| temporal | L | -54 | -22 | 9 | inferior cerebellum | L | -34 | -67 | -29 | 0.70 | 10.04 |
| **Parietal-Parietal** | | | | | | | | | | | |
| inferior parietal lobule | R | 54 | -44 | 43 | superior parietal | R | 34 | -39 | 65 | 0.72 | 9.71 |
| inferior parietal lobule | R | 54 | -44 | 43 | parietal | R | 41 | -23 | 55 | 0.72 | 9.35 |
| inferior parietal lobule | R | 54 | -44 | 43 | parietal | R | 46 | -20 | 45 | 0.71 | 9.59 |
| inferior parietal lobule | R | 44 | -52 | 47 | superior parietal | R | 34 | -39 | 65 | 0.71 | 9.61 |
| **Others** |  |  |  |  |  |  |  |  |  |  |  |
| parietal | L | -47 | -12 | 36 | dorsal frontal cortex | R | 60 | 8 | 34 | -0.78 | -8.69 |
| inferior temporal | L | -61 | -41 | -2 | middle insula | R | 33 | -12 | 16 | -0.72 | -11.50 |
| inferior temporal | L | -61 | -41 | -2 | temporal | R | 59 | -13 | 8 | -0.70 | -10.26 |
| inferior temporal | R | 52 | -15 | -13 | angular gyrus | R | 51 | -59 | 34 | 0.72 | 11.45 |

**References**

1 Nahum M, Fisher M, Loewy R, Poelke G, Ventura J, Nuechterlein KH *et al.* A novel, online social cognitive training program for young adults with schizophrenia: A pilot study. *Schizophr Res Cogn* 2014; **1**: e11–e19.

2 Fisher M, Nahum M, Howard E, Rowlands A, Brandrett B, Kermott A *et al.* Supplementing intensive targeted computerized cognitive training with social cognitive exercises for people with schizophrenia: An interim report. *Psychiatr Rehabil J* 2017; **40**: 21–32.

3 Cohen J. *Statistical power analysis for the behavioral sciences*. 2nd ed. Lawrence Erlbaum Associates: New Jersey, NJ, 1988 doi:10.4324/9780203771587.

4 Nowicki S, Duke MP. Individual differences in the nonverbal communication of affect: The diagnostic analysis of nonverbal accuracy scale. *J Nonverbal Behav* 1994; **18**: 9–35.

5 Wechsler D. Adult intelligence scale—3rd edition (WAIS-3R). 1997.

6 Strauss E, Sherman EM, Spreen O. *A compendium of neuropsychological tests: Administration, norms, and commentary*. American Chemical Society, 2006.

7 Lezak MD, Howieson DB, Loring DW. Neuropsychological assessment. New York: Oxford Univer. *Press Google Scholar* 1995.

8 Rey A. L’examen psychologique dans les cas d'encéphalopathie traumatique.(Les problems.). *Arch Psychol (Geneve)* 1941.

9 Reitan RM. Trail making test: manual for administration and scoring [adults]: Reitan Neuropsychology Laboratory. *Tucson, Ariz* 1992.

10 Cornblatt BA, Risch NJ, Faris G, Friedman D, Erlenmeyer-Kimling L. The Continuous Performance Test, identical pairs version (CPT-IP): I. New findings about sustained attention in normal families. *Psychiatry Res* 1988; **26**: 223–238.

11 Petrides M, Milner B. Deficits on subject-ordered tasks after frontal- and temporal-lobe lesions in man. *Neuropsychologia* 1982; **20**: 249–262.

12 Nuechterlein KH, Green MF, Kern RS, Baade LE, Barch DM, Cohen JD *et al.* The MATRICS Consensus Cognitive Battery, part 1: test selection, reliability, and validity. *Am J Psychiatry* 2008; **165**: 203–213.

13 Manjón JV, Carbonell-Caballero J, Lull JJ, García-Martí G, Martí-Bonmatí L, Robles M. MRI denoising using non-local means. *Med Image Anal* 2008; **12**: 514–523.

14 Rajapakse JC, Giedd JN, Rapoport JL. Statistical approach to segmentation of single-channel cerebral MR images. *IEEE Trans Med Imaging* 1997; **16**: 176–186.

15 Patel AX, Kundu P, Rubinov M, Jones PS, Vértes PE, Ersche KD *et al.* A wavelet method for modeling and despiking motion artifacts from resting-state fMRI time series. *Neuroimage* 2014; **95**: 287–304.

16 Power JD, Barnes KA, Snyder AZ, Schlaggar BL, Petersen SE. Spurious but systematic correlations in functional connectivity MRI networks arise from subject motion. *Neuroimage* 2012; **59**: 2142–2154.

17 Power JD, Mitra A, Laumann TO, Snyder AZ, Schlaggar BL, Petersen SE. Methods to detect, characterize, and remove motion artifact in resting state fMRI. *Neuroimage* 2014; **84**: 320–341.

18 Satterthwaite TD, Elliott MA, Gerraty RT, Ruparel K, Loughead J, Calkins ME *et al.* An improved framework for confound regression and filtering for control of motion artifact in the preprocessing of resting-state functional connectivity data. *Neuroimage* 2013; **64**: 240–256.

19 Song X-W, Dong Z-Y, Long X-Y, Li S-F, Zuo X-N, Zhu C-Z *et al.* REST: a toolkit for resting-state functional magnetic resonance imaging data processing. *PLoS One* 2011; **6**: e25031.

20 Ruschhaupt M, Huber W, Poustka A, Mansmann U. A compendium to ensure computational reproducibility in high-dimensional classification tasks. *Stat Appl Genet Mol Biol* 2004; **3**: Article37.

21 Koutsouleris N, Kahn RS, Chekroud AM, Leucht S, Falkai P, Wobrock T *et al.* Multisite prediction of 4-week and 52-week treatment outcomes in patients with first-episode psychosis: a machine learning approach. *Lancet Psychiatry* 2016; **3**: 935–946.

22 Dwyer DB, Falkai P, Koutsouleris N. Machine learning approaches for clinical psychology and psychiatry. *Annu Rev Clin Psychol* 2018; **14**: 91–118.

23 Filzmoser P, Liebmann B, Varmuza K. Repeated double cross validation. *J Chemom* 2009; **23**: 160–171.

24 Cabral C, Kambeitz-Ilankovic L, Kambeitz J, Calhoun VD, Dwyer DB, von Saldern S *et al.* Classifying schizophrenia using multimodal multivariate pattern recognition analysis: evaluating the impact of individual clinical profiles on the neurodiagnostic performance. *Schizophr Bull* 2016; **42 Suppl 1**: S110–7.

25 Hansen LK, Larsen J, Nielsen FA, Strother SC, Rostrup E, Savoy R *et al.* Generalizable patterns in neuroimaging: how many principal components? *Neuroimage* 1999; **9**: 534–544.

26 Vapnik VN. An overview of statistical learning theory. *IEEE Trans Neural Netw* 1999; **10**: 988–999.

27 Fan Y, Gur RE, Gur RC, Wu X, Shen D, Calkins ME *et al.* Unaffected family members and schizophrenia patients share brain structure patterns: a high-dimensional pattern classification study. *Biol Psychiatry* 2008; **63**: 118–124.

28 Golland P, Fischl B. Permutation tests for classification: towards statistical significance in image-based studies. *Inf Process Med Imaging* 2003; **18**: 330–341.

29 Koutsouleris N, Kambeitz-Ilankovic L, Ruhrmann S, Rosen M, Ruef A, Dwyer DB *et al.* Prediction Models of Functional Outcomes for Individuals in the Clinical High-Risk State for Psychosis or With Recent-Onset Depression: A Multimodal, Multisite Machine Learning Analysis. *JAMA Psychiatry* 2018; **75**: 1156–1172.

30 Xia M, Wang J, He Y. BrainNet Viewer: a network visualization tool for human brain connectomics. *PLoS One* 2013; **8**: e68910.
